# Supplementary material for: An automation framework for clinical codelist development validated with UK data from patients with multiple long-term conditions
Source: BMC Med Res Methodol. 2025 May 24;25:138. doi: 10.1186/s12874-025-02541-1 (PMC12102889; doi:10.1186/s12874-025-02541-1)
Supplement: Supplementary file 1 — Supplementary Material 1. [file 12874_2025_2541_MOESM1_ESM.zip › Codelist_Paper_BMC_Appendix.pdf]

Appendix A DynAIRx Codelist Stats

Please find the list conditions covered by the DynAIRx codelist in Table A1 using the Generalised Codelist Automation Framework (GCAF). All codelists including additional conditions can be found in attached repository of supplementary material.

Table A1: All conditions covered by the DynAIRx codelist.

**DynAIRx Codelist Condition Names**			
Acne	Dizziness	Myasthenia Gravis	Social vulnerability
Activity limitation	Down Syndrome	Obstructive Sleep Apnoea	Spina bifida
Alcoholic Liver Disease	Dressing &grooming problems	Occupational lung diseases	Spinal stenosis
Alcohol-related Brain Injury	Dysmenorrhoea	Oesophageal varices	Splenomegaly
Anaemia B12 Deficiency	Dyspnoea	Osteoarthritis	Spondylolisthesis
Anaemia Folate Deficiency	End Stage Renal Disease	Osteoporosis	Spondylosis
Anaemia Haemolytic	Endometriosis	Palliative care	Systemic Sclerosis
Anaemia Iron Deficiency	Environment problems	Parkinson and Tremor	Thalassaemia
Anaemia Other	Erectile Dysfunction	Peptic ulcer Disease	Thrombophilia
Anorectal Prolapse	Faecal incontinence	Peripheral Neuropathies	Thyroid Problem
AnorexiaBulimia	Falls	Personality Disorder	Toileting problems
Anterior and Intermediate Uveitis	Fatty Liver	Pituitary adenoma	Urinary system disease
Anxiety	Female genital Prolapse	Polycystic Ovarian Syndrome	Urinary_Incontinence
Asbestosis	Fibroids	Polycythaemia vera	Urolithiasis
Asthma	Foot problems	Polymyalgia Rheumatica	Vitiligo
Autoimmune liver Disease	Fracture	Primary Idiopathic Thrombocytopaenia	Washing & bathing problems
Autonomic dysfunction	Fragility fracture	Primary Pulmonary hypertension	Weakness
Back pain	Gastritis and Duodenitis	Problems managing finances	Weight loss
Benign	Glaucoma	Prostate Disorder	Abdominal Aortic Aneurysm
Bipolar	Gout	Psoriatic Arthropathy	Abdominal Hernia
Bladder Dysfunction	Headache	Psychoactive Substance Misuse	Actinic keratosis
Bone disease	Hearing Loss	Pulmonary Fibrosis	Allergic And Chronic rhinitis
Bronchiectasis	Heart Failure	Pulmonary hypertension	Ankylosing Spondylitis
Cancer	Heart block	Raynaud Syndrome	Attention Deficit Hyperactivity Disorder
Cancer Haematological	HIV	Renal and Bladder Stones	Autism and Asperger Syndrome
Cancer Solid organ	Housebound	Renal Stones	Barrett Oesophagus
Cataract	Hyperparathyroidism	Requirement for care	Carcinoma in situ Cervical

**DynAIRx Codelist Condition Names**			
Cerebral palsy	Hypertension	Respiratory failure	Chronic Tinnitus
Cholelithiasis	Hypertrophic Cardiomyopathy	Rheumatic heart disease	Colonic polyp
Chronic Dermatitis Eczema	Hyposplenism	Rheumatoid Arthritis	Endometrial Hyperplasia and Hypertrophy
Chronic Obstructive Pulmonary Disease (COPD)	Hypotension syncope	Rosacea	Gastrooesophageal Reflux Disease
Chronic Pancreatitis	Immunodeficiencies	Sarcoidosis	Inflammatory arthritis
Chronic Sinusitis	Interstitial lung disease	Schizoaffective	Learning Disability
Chronic Urticaria	Liver Disease - Other	Scoliosis	Menorrhagia and Polymenorrhea
Chronic constipation	Liver Disease - Viral	Secondary Polycythaemia	Multiple Sclerosis
CKD	Lupus Erythematosus	Seizure Disorders	OCD
Coeliac Disease	Macular Disorders	Self-harm	Psoriasis
Complex pain syndrome	Meal preparation problems	Shopping problems	Skin ulcer
Cystic fibrosis	Medication management problems	Sick sinus Syndrome	Stress
Dementia	Meniere Disease	Sickle cell anaemia	Subdural Haematoma no-traumatic
Depression	Migraine	Sjogren Disease	Uterovaginal Genital Prolapse
Dermatitis atopic contact	Mobility problems	Sleep apnoea	
Dilated Cardiomyopathy	Motor Neurone Disease	Sleep problems	
Diverticular Disease	Musculoskeletal problems	Smoking	
